# *Eurotium cristatum*-Fermented White Tea Ameliorates DSS-Induced Colitis by Multi-Scale

**DOI:** 10.3390/foods15010072

**Published:** 2025-12-25

**Authors:** Huini Wu, Xiangrui Kong, Ruiyang Shan, Song Peng, Mengshi Zhao, Wenquan Yu, Changsong Chen, Xiuping Wang, Zhaolong Li

**Affiliations:** 1 Institute of Subtropical Agriculture, Fujian Academy of Agricultural Sciences, Zhangzhou 363005, China; wuhuinifaas@163.com; 2 Institute of Animal Husbandry and Veterinary Medicine, Fujian Academy of Agricultural Sciences, Fuzhou 350013, China; pengsong@faas.cn (S.P.); 13375001253@163.com (M.Z.); 3 Tea Research Institute, Fujian Academy of Agricultural Sciences, Fuzhou 350013, China; kongxiangrui_2008@163.com (X.K.); fjnkycys@163.com (R.S.); yuwq@faas.cn (W.Y.); ccs6536597@163.com (C.C.)

**Keywords:** *Eurotium cristatum*, fermentation, white tea, DSS, Single-cell and spatial transcriptomic, colon barrier

## Abstract

*Eurotium cristatum*-Fermented White Tea (FWT) significantly alters white tea (WT) composition, increasing caffeine while decreasing polyphenols and amino acids. FWT effectively ameliorated dextran sulfate sodium (DSS)-induced murine colitis symptoms (reducing weight loss, colon shortening). Mechanistically, FWT suppressed TLR4/Myd88/NF-κB signaling and pro-inflammatory cytokines (TNF-α, IL-6, IL-1β) while upregulating tight junction proteins (ZO-1, occludin, claudin-1), MUC2, and E-cadherin. Single-cell/spatial transcriptomics revealed that FWT treatments augment enterocyte, goblet cell, and stem cell populations, optimize goblet function, restructure stem cell differentiation, and induce epithelial REG3B (antimicrobial) and LYPD8 (motility inhibitor), plus immunomodulator GM42418 lncRNA across cell types, repairing the barrier. FWT intervention was also associated with an increase in beneficial bacteria (*Akkermansia*, *Lactobacillus*, *Bifidobacterium*), restoration of microbiota balance, and elevated levels of short-chain fatty acids (SCFAs) and was associated with alterations in caffeine-related metabolite profiles. Collectively, these multi-scale changes correlate with the alleviation of UC, suggesting an integrated mechanism involving mucosal barrier repair, immune–stromal modulation, microbiota–metabolism regulation, and cellular reprogramming.

## 1. Introduction

Ulcerative colitis (UC), a primary inflammatory bowel disease (IBD), manifests as weight loss, bloody stools, diarrhea, and abdominal pain. Its pathogenesis, while incompletely understood, involves intestinal mucosal barrier damage, immune system disorders, and gut flora imbalance, influenced by diet, environment, and heredity [1,2]. Recent advances in single-cell transcriptomics (scRNA-seq) reveal UC pathology through cellular heterogeneity, epithelial–stromal reprogramming, immune dysregulation (including pathogenic macrophages), and neuro–immune interactions driving barrier breakdown [3]. The current therapeutic armamentarium for UC includes 5-aminosalicylic acid (5-ASA) derivatives, corticosteroids, immunomodulators (azathioprine), biologic agents (such as anti-TNF antibodies, vedolizumab, and ustekinumab), and small-molecule drugs (Janus kinase inhibitors) [4,5]. Despite these available options, many patients still exhibit a suboptimal therapeutic response, secondary loss of efficacy, or unacceptable adverse events, underscoring the persistent demand for safer and more effective treatment strategies.

Damage to the intestinal mucosal barrier, characterized by impaired tight junction proteins and reduced mucin 2 (MUC2) expression, increases permeability. This allows pathogens and their products to penetrate, triggering persistent inflammation [6]. Inflammation stimulates pro-inflammatory cytokines, such as interleukin-1β (IL-1β), interleukin-6 (IL-6), and tumor necrosis factor-α (TNF-α), thereby exacerbating intestinal inflammation. Furthermore, the abnormal activation of inflammation-related signaling pathways, including myeloid differentiation factor 88 (MyD88), nuclear factor-κB (NF-κB), and toll-like receptor 4 (TLR4), can further stimulate the production of numerous inflammatory factors, aggravating UC [7,8]. TNF-α and TLR4 also promote inducible nitric oxide synthase 2 (iNOS2) expression, leading to excessive nitric oxide (NO) that worsens inflammation [9]. Conversely, anti-inflammatory cytokines like interleukin-10 (IL-10) help maintain immune balance. The intestinal flora, vital for digestion and vitamin synthesis and inhibiting pathogens via competition, reduces inflammation [10]. In UC, dysbiosis occurs—marked by *Firmicutes*/*Bacteroidetes* imbalance, decreased beneficial bacteria (*Bifidobacterium*, *Lactobacillus*, *Akkermansia*), and increased *Enterobacteriaceae*. This disrupts the mucosal barrier, impairs mucosal immunity, and exacerbates UC [11]. Thus, the mucosal barrier, immune system, and microbiota are critical interconnected components in UC pathogenesis.

Tea, produced from the leaves of *Camellia sinensis* (family *Theaceae*), a globally consumed beverage rich in bioactive components (polyphenols, catechins, caffeine), exhibits antioxidant, anti-inflammatory, and antibacterial properties [12,13]. Studies demonstrate that its active ingredients ameliorate DSS-induced colitis in mice [14,15,16]. Pu’er tea enhances intestinal barrier function by modulating microbiota (increasing *Lactobacillus*, *Akkermansia*, and *Faecalis* while reducing pathogens), upregulating TJ proteins, regulating inflammation (decreasing IL-1β and IL-6 while increasing IL-10 and interleukin-22 (IL-22) via PI3K/AKT/NF-κB pathway modulation), and elevating SCFAs [17]. Fuzhuan brick tea polysaccharide (FBTPS) reduces serum IL-6, IL-1β, interferon-gamma (IFN-γ), TNF-α, and lipopolysaccharide (LPS); suppresses inflammation-related mRNA; enhances Occludin, Claudin-1, and zonula occludens-1 (ZO-1) expression; mitigates goblet cell loss; promotes probiotics (*Bacteroides*, *Parabacteroides*, *Collinsella*); and increases SCFAs [18]. White tea (WT) components (catechins, alkaloids, amino acids, and phenolic acids) neutralize ROS, inhibit harmful bacteria (*g-Bacteroides*, *g-Escherichia-Shigella*), promote beneficial bacteria, and upregulate SCFAs, bile acids, and amino acids to alleviate UC [19]. Beyond tea, various other natural products have shown beneficial effects in animal models of colitis, primarily by modulating the gut microbiota and enhancing intestinal barrier function. Examples include specific flavonoids from plants like *Penthorum chinense* Pursh, polyphenols from *Artemisia argyi*, and microbial metabolites such as isobutyrate, which can alleviate colitis by restoring microbial balance, increasing SCFAs, and regulating inflammatory pathways [20,21,22]. Research has extended the application of *Eurotium cristatum*, a key fungus from Fuzhuan brick tea, to the fermentation of other tea types (e.g., green tea and Pu’er tea), demonstrating its broad impact on quality and functionality [23,24]. However, the potential effects of WT fermented by *Eurotium cristatum* (EC-520) on UC and the underlying mechanisms remain unclear. Consequently, this study investigated FWT’s impact on DSS-induced UC histopathology, clinical manifestations, intestinal mucosal barrier function, gut microbiota–metabolite interactions, and cellular–spatial reprogramming. We elucidated dose-dependent epithelial remodeling, immune–stromal modulation, and neuro–immune crosstalk to reveal FWT’s multi-scale mechanisms alleviating colitis from molecular to spatial niches, thereby providing new insights and a mechanistic basis for the future development of UC therapeutic strategies and fermented tea applications.

## 2. Materials and Methods

### 2.1. Chemicals and Reagents

Dextran sulfate sodium salt (DSS, molecular weight 36–50 kDa) was obtained from MP Biomedicals (Irvine, CA, USA). The IL-1β, TNF-α, IL-6, and IL-10 ELISA kits were purchased from Thermo Fisher Scientific Inc.(Wuhan, China). Quantitative PCR primers for TNF-α, IL-1β, iNOS2, occludin, ZO-1, MUC2, claudin-1, TLR4, MyD88, and NF-κB were synthesized by Sunya Biotechnology Co., Ltd. (Fuzhou, China). Standards for acetic acid, propionic acid, butyric acid, valeric acid, hexanoic acid, isobutyric acid, and 2-methylbutyric acid were obtained from Zhenzhun Biotechnology Co., Ltd. (Shanghai, China). Anti-ZO-1, anti-claudin-1 (1:500), anti-MUC2 (1:200), anti-occludin (1:500), anti-Epithelial-cadherin (E-cadherin, 1:500), goat anti-rabbit IgG (1:300), and 4′,6-diamidino-2-phenylindole (DAPI) were purchased from Servicebio Technology Co., Ltd. (Wuhan, China). All other reagents were analytical grade.

### 2.2. Tea Sample Preparations and Chemical Characterization Assays

White tea (WT) raw materials (2016 production) were sourced from Huaxiangyuan Tea Industry (Xiamen, China). These materials were processed from the *Camellia sinensis* cv. ‘Fuding Dabai’. The tender shoots, comprising one apical bud and two or three young leaves, were harvested from plantations in the Wuyi Mountain region (Fujian province), a cultivation area characterized by an altitude of 600–800 m, a humid subtropical climate with a mean annual temperature of 18–18.5 °C and an average relative humidity of approximately 80%, and acidic red loam soils. The EC-520 strain, isolated from FBT with specific genetic traits [25], was used for fermentation. WT (800 g) was sterilized (120 °C, 10 min), cooled, and inoculated with EC-520 suspension (1 × 10^8^ CFU/mL) under sterile conditions. Fermentation proceeded at 28 °C for 7 days. Fermented white tea (FWT) was dried (45 °C, 8 h). Both WT and FWT were ground, extracted twice with boiling pure water (1:15 then 1:10, *w*/*v*; 30 min each), filtered, combined, freeze-dried, and stored at −80 °C. FWT and WT extracts were analyzed by LC-MS for major metabolites. The raw mass spectrometry data were preprocessed sequentially: outliers were filtered out based on interquartile range; peaks with missing values exceeding 50% within any experimental group or across all samples were removed; remaining missing values were imputed using half of the minimum value; and all peak responses were normalized against the internal standard peak area.

Water content was determined by weight difference before/after heating at 103 °C. Polyphenols (Folin–Ciocalteu), caffeine (HPLC), free amino acids (ninhydrin), and total soluble sugar content were determined using the 3,5-dinitrosalicylic acid (DNS) colorimetric assay.

### 2.3. Animal Experiments

Female C57BL/6 mice (7−8 weeks, 18−20 g) were obtained from Wu’s Laboratory Animal Co., Ltd. (Fuzhou, China) and housed under standard conditions with access to sterile water and a basic diet for a 7-day acclimatization period. The animal care and use protocol was approved by the Institutional Animal Care and Use Committee at the Institute of Animal Husbandry and Veterinary Medicine of Fujian Academy of Agricultural Sciences (202307FJ010). Subsequently, 60 mice were randomly divided into six groups (*n* = 10): (1) normal control group (CK); (2) DSS-induced model group (DSS); (3) DSS + high dose FWT group (HFWT); (4) DSS + low dose FWT group (LFWT); (5) DSS + high dose WT group (HWT); and (6) DSS + low dose WT group (LWT). The DSS, HFWT, LFWT, HWT, and LWT groups received 3% DSS in drinking water from days 1 to 7, while the CK group received normal water. From days 8 to 14, the HFWT and LFWT groups were administered FWT by gavage at 400 and 200 mg/kg·(body weight)/day, respectively, while the HWT and LWT groups received WT at corresponding doses, based on established protocols [18,19]. The CK and DSS groups received sterile water. Body weight, stool consistency, and rectal bleeding were monitored daily. On day 15, mice were euthanized, and serum, colon, and fecal samples were collected for subsequent analyses.

### 2.4. Evaluation of the DAI

The Disease Activity Index (DAI) for assessing the degree and severity of UC was evaluated through a combination of scores. In accordance with the existing literature, the evaluation system based on body weight loss, stool consistency, and rectal bleeding is presented in Table 1.

### 2.5. Histopathological Analysis

Colon tissues were fixed in 4% paraformaldehyde, paraffin-embedded, sectioned, deparaffinized in xylene, and rehydrated. For H&E staining, sections were stained with hematoxylin (1–3 min), differentiated in acetic acid, blued in ammonia, counterstained with eosin (1–2 min), dehydrated, cleared in xylene, and mounted. For AB-PAS staining, sections were stained with Alcian Blue (30 min), rinsed, oxidized in periodic acid (5 min), rinsed, treated with Schiff’s reagent (10–20 min), counterstained with hematoxylin (1 min), dehydrated, cleared, and mounted. Stained sections were examined by light microscopy (OLYMPUS, Tokyo, Japan). Colonic tissue was scored as previously described [26], and goblet cells per unit area were quantified using ImageJ software (version 1.54i; NIH, Bethesda, MD, USA & LOCI, Madison, WI, USA).

### 2.6. Immunofluorescence Analysis

Colon tissue was fixed in acetone at −20 °C and subsequently incubated overnight at −4 °C with primary antibodies (claudin-1, occludin, ZO-1, MUC2, and E-cadherin). Following three washes with PBS, the slices were treated with goat anti-rabbit IgG at room temperature in the absence of light for 50 min. After three additional washes with PBS, the sections were re-stained with DAPI. The images obtained were quantitatively analyzed using ImageJ software.

### 2.7. Determination of Serum Cytokines

Serum levels of IL-6, IL-10, TNF-α, and IL-1β were measured using a commercial ELISA kit (Thermo Fisher Scientific Inc., Wuhan, China) following the manufacturer’s instructions. The optical density (OD) at 450 nm was recorded using an enzyme-linked immunoassay analyzer. The quadratic regression equation for the standard curve was calculated based on the OD values of the standards, with concentration as the ordinate. The serum levels of TNF-α, IL-1β, IL-6, and IL-10 in each group were determined by applying the standard curve to the OD values of the samples.

### 2.8. Determination of SCFAs in the Colon Fecal

Fecal samples were homogenized in 80% methanol and centrifuged (12,000 rpm, 10 min) to deproteinize. The supernatant was derivatized with 150 μL reagent (40 °C, 40 min), diluted with 80% methanol, and then mixed with 5 μL internal standard per 95 μL supernatant. SCFAs were quantified using UHPLC-MS/MS (Thermo Vanquish™ Flex-TSQ Altis™) at BIOZERON (Shanghai, China). Chromatography used a Waters BEH C18 column (2.1 × 100 mm, 1.7 μm; 40 °C) with mobile phase: A) 10 mM ammonium acetate/water; B) acetonitrile/isopropanol (1:1); flow 0.30 mL/min. Gradient: 25% B (2.5 min), then stepwise increases to 30% (3 min), 35% (3.5 min), 38% (4 min), 40% (4.5 min), 45% (5 min), 50% (5.5 min), 55% (6.5 min), 58% (7 min), 70% (7.5 min), 100% (7.8 min), back to 25% (10.1 min), and is held for 12 min. MS operated in negative MRM mode: IonSpray Voltage −4500 V, Sheath Gas 35 psi, Source Temp 550 °C, Aux Gas 50 psi, Collision Gas 55 psi.

### 2.9. Gut Microbiota Analysis

Fresh fecal samples were stored at −80 °C and subsequently sent to BIOZERON Biotechnology Co., Ltd. (Shanghai, China) for 16S rDNA sequencing analysis. Total genomic DNA was extracted from the fecal samples following the instructions provided with the DNA extraction kit. The V3-V4 region of the 16S rRNA gene was then amplified using the primers 338F (5′-ACTCCTACGGGAGGCAGCAG-3′) and 806R (5′-GGACTACHVGGGTWTC TAAT-3′). The purified amplification product (amplicon) was analyzed using the NovaSeq 600 platform (Illumina, San Diego, CA, USA). Operational Taxonomic Units (OTUs) were clustered with a similarity cutoff of 98.65% using UPARSE (version 7.1, http://drive5.com/uparse/, accessed on 15 March 2025), and chimeric sequences were identified and removed using UCHIME. All results, including alpha diversity, beta diversity, and microbial community structures, were based on the features of the OTUs. Five mice were used per group (*n* = 5), and no pooling of samples was performed. Alpha diversity indices (Chao1 and Shannon) were compared among groups using the Kruskal–Wallis test, with significance set at *p <* 0.05. Beta diversity was assessed by principal coordinate analysis (PCoA) based on Bray–Curtis distance. Differentially abundant microbial taxa were identified using LEfSe analysis (LDA score > 3.5).

### 2.10. HPLC-MS Analysis for Colon Fecal

One hundred milligrams of fecal material was ground in liquid nitrogen. The homogenate was vortexed in prechilled 80% methanol/0.1% formic acid, incubated on ice (5 min), and centrifuged (15,000 rpm, 5 min, 4 °C). The supernatant was diluted to 53% methanol with water and recentrifuged (15,000 g, 10 min), and the final supernatant was analyzed. LC-MS/MS used a Thermo Fisher Vanquish UHPLC coupled to a Q Exactive™ HF mass spectrometer (BIOZERON Biotechnology Co., Ltd., Shanghai, China). Analysis occurred in ESI positive and negative modes. Chromatography used a Hypersil Gold C18 column (100 × 2.1 mm, 1.9 μm; 45 °C) with 2 μL injection. Mobile phase: (A) 0.1% formic acid/water; (B) methanol; flow 0.2 mL/min. Gradient: 2% B (1.5 min), 2–100% B (12.0 min), 100% B (14.0 min), 100–2% B (0.1 min), 2% B (3 min). MS settings: spray voltage 3.2 kV, capillary 320 °C, sheath gas 40 arb, auxiliary gas 10 arb. Metabolomics data were processed by peak extraction, alignment, and normalization. Differential metabolites were screened with the thresholds: |log_2_FC| > 1, variable importance in projection (VIP) > 1, and *p <* 0.05. Orthogonal partial least squares–discriminant analysis (OPLS-DA) was applied to evaluate group separation. KEGG pathway enrichment analysis was performed using a hypergeometric test, with *p <* 0.05 considered significantly enriched.

### 2.11. Quantitative Analysis of the Colon Gene Expression

Total RNA was extracted from colon tissue using an RNA extraction kit. RNA concentration/purity was assessed with a BioSpec-nano UV spectrophotometer. RNA was reverse-transcribed into cDNA using PrimeScript RT Master Mix. Quantitative reverse transcription polymerase chain reaction (qRT-PCR) was performed with SYBR Green Master Mix on a CFX Connect instrument, using Glyceraldehyde-3-phosphate dehydrogenase (GAPDH) as the internal reference. Target gene expression was calculated via the 2^−ΔΔCt^ method. Primers (sequences in Table 2) were synthesized by Sunya Biotechnology (Fuzhou, China). All steps followed the manufacturers’ protocols.

### 2.12. Single-Nucleus RNA Sequencing (snRNA-Seq)

snRNA-seq was performed on colon tissues from three mice per experimental group (*n* = 3). A 1 cm mid-colon segment underwent rapid immersion in OCT compound (Sakura Finetek, Tokyo, Japan), snap-freezing in liquid nitrogen, and storage at −80 °C. Following a 2 h equilibration at −20 °C, tissues were cryosectioned at 30 μm thickness using a cryostat (Leica CM1950, Wetzlar, Germany). Sections were transferred to prechilled nuclear isolation buffer (0.1% NP-40, 1 mM DTT, 0.4 U/μL RNase inhibitor [Takara], 1% BSA [Sigma-Aldrich, St. Louis, MO, USA] in DPBS). After mechanical homogenization and filtration through a 40 μm cell strainer, intact nuclei (DAPI-positive, 5–15 μm diameter) were flow-sorted (BD FACSAria^TM^III, BD Biosciences, NJ, USA). Nuclear suspensions were adjusted to 700–1000 nuclei/μL. Libraries were constructed per the 10× Genomics Single Cell 3′ RNA-Seq v3.1 Kit protocol and sequenced on an Illumina NovaSeq 6000 platform (150 bp paired-end reads). Nuclear viability (>85%) was confirmed by Trypan Blue staining (Thermo Fisher). Raw data were aligned to the mm10 genome using Cell Ranger v6.1.2 (10× Genomics, CA, USA) for gene expression. Subsequent analysis in Seurat v4.3.0 included quality control (nuclei with >500 UMIs, mitochondrial genes <10%), normalization, graph-based clustering, and differential expression analysis (|log2FC| > 0.5, FDR < 0.05) to compare transcriptional profiles across subpopulations. Nuclei from each sample were sequenced with a target of 5000 nuclei per replicate and a median sequencing depth of 20,000 reads per nucleus. Batch effects were mitigated using Seurat’s IntegrateData function. Nuclear types were annotated based on established marker genes (e.g., Fabp1 for enterocytes, Muc2 for goblet cells, Lgr5 for stem cells). Differential cell abundance across groups was assessed using a pseudobulk approach with DESeq2, and compositional effects were accounted for in statistical modeling.

### 2.13. Spatial Transcriptomics Analysis

Spatial transcriptomics was performed on colon sections from the same three mice per group used for snRNA-seq (*n* = 3). Mid-colon segments (0.5 cm) were OCT-embedded, sectioned at 10 μm, and mounted on Visium Spatial Gene Expression Slides (10× Genomics, CA, USA). Sections were fixed in −20 °C methanol (Sigma-Aldrich) for 5 min, H&E-stained (Abcam, Cambridge, UK), and imaged microscopically. Permeabilization duration, optimized to 12 min with the Visium Tissue Optimization Kit (10× Genomics, CA, USA), ensured efficient mRNA release and probe capture. Using the Visium Spatial Gene Expression Reagent Kit, libraries were constructed and sequenced via Illumina NovaSeq 6000 (150 bp paired-end reads). Each section yielded 3000–5000 spatially barcoded spots. Space Ranger v2.0.0 (10× Genomics, CA, USA) processed the raw data, aligning transcripts to the tissue morphology using H&E images and enforcing a quality threshold (>5000 unique molecular identifiers per spot). To resolve cell-type composition per spot, SPOTlight v0.1.7 deconvoluted the data, integrating prior snRNA-seq clustering results. Spatially resolved differential gene expression analysis was then conducted to map regionalized colonic cellular subpopulations. Cell-type deconvolution was performed using SPOTlight with snRNA-seq clusters as reference; spatial assignments were validated by overlapping with canonical marker gene expression (e.g., Muc2 for goblet cells, Lgr5 for stem cells). Spatially resolved differential gene expression analysis was conducted to identify region-specific cellular subpopulations. Changes in spatial cell distribution across groups were assessed using linear mixed-effects models that accounted for intra-sample correlation, with non-parametric alternatives (Wilcoxon rank-sum test) applied where assumptions were not met.

### 2.14. Statistical Analysis

Statistical analyses were conducted using SPSS version 26.0. Data are presented as mean ± standard error of the mean (SEM). The *t*-test was employed for comparisons between two groups, whereas one-way ANOVA was utilized for comparisons among multiple groups. A *p*-value of less than 0.05 was deemed statistically significant. Additionally, GraphPad Prism version 9.4.0 was used for data plotting.

## 3. Results

### 3.1. Chemical Composition of FWT and WT Extracts

**Figure 1:**
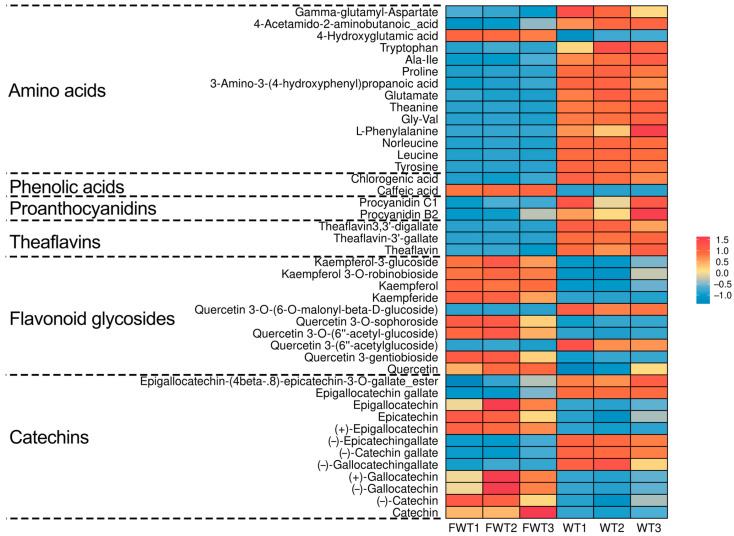
Heat map of the contents of 43 significantly differential compounds in FWT and WT extracts.

To analyze the phytochemicals in FWT and WT, an LC-MS-based nontargeted metabolomics approach identified 1445 compounds (Table S1). We focused on 43 key nonvolatile tea-related constituents, including catechins, flavonoid glycosides, theaflavins, proanthocyanidins, phenolic acids, and amino acids. The selection of these 43 key tea-related constituents was based on their established biological relevance and frequent reporting in the literature on tea phytochemistry and bioactivity, allowing for a targeted analysis of fermentation-induced changes [27,28]. Their distributions are shown in Figure 1. Additionally, contents of tea polyphenols, soluble sugar, water extract, caffeine, and free amino acids were measured (Table 3). In FWT, water extract, tea polyphenols, amino acids, proanthocyanidins, and theaflavins decreased significantly, while caffeine increased. Levels of gallocatechin gallate (GCG), catechin gallate (CG), and epigallocatechin gallate (EGCG) were down-regulated, whereas catechin (C), gallocatechin (GC), epigallocatechin (EGC), epicatechin (EC), and most flavonoid glycosides increased. These changes are likely due to metabolic activities of *Eurotium cristatum* during fermentation. It should be noted that the animal interventions were conducted based on equal mass doses of the tea extracts (mg extract per kg body weight). Therefore, the observed biological differences between FWT and WT groups may stem from the combined effects of their distinct compositional profiles (e.g., higher caffeine but lower polyphenols in FWT) resulting from fermentation, rather than from a difference in the administered quantity of raw material. Future studies employing dose-matching strategies for specific key components (e.g., adjusting WT dose to match the caffeine content in FWT) would be valuable to delineate the contribution of individual constituents.

### 3.2. FWT Relieved the Symptoms of DSS-Induced UC

**Figure 2:**
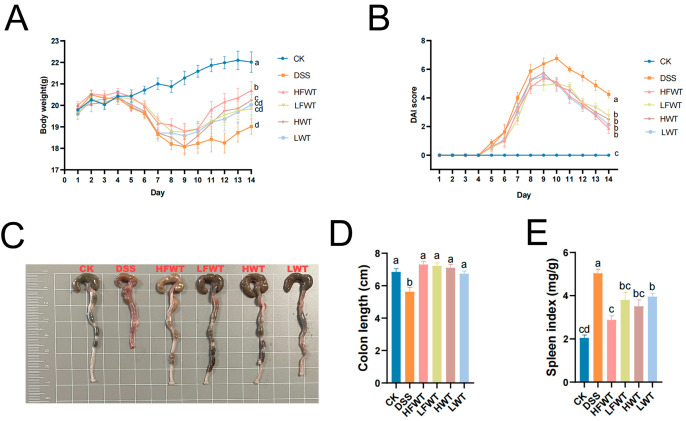
FWT alleviated DSS-induced colitis symptoms. (**A**) Body weight; (**B**) DAI score; (**C**) representative colon images; (**D**) colon length; (**E**) spleen index. Data are mean ± SEM (*n* = 8). FWT, *Eurotium cristatum*-Fermented White Tea; WT, White Tea; CK, normal control group; DSS, DSS-induced model group; HFWT, DSS + high-dose FWT group; LFWT, DSS + low-dose FWT group; HWT, DSS + high-dose WT group; LWT, DSS + low-dose WT group. Letters a–d indicate significant differences (*p <* 0.05).

As shown in Figure 2A,B, the body weights of the mice in the experimental groups were lower, and the Disease Activity Index (DAI) scores were significantly higher compared to the CK group during the DSS treatment. Following the cessation of DSS treatment, both body weight and DAI scores began to recover gradually. Notably, the HFWT, LFWT, HWT, and LWT groups exhibited a more pronounced recovery trend compared to the DSS group. Additionally, these groups significantly mitigated (*p <* 0.05) the symptoms of colon length shortening and spleen index increase induced by DSS, as illustrated in Figure 2C–E. These results indicated that both FWT and WT interventions could alleviate symptoms in DSS-induced mice, with HFWT demonstrating the highest efficacy (*p <* 0.05).

### 3.3. FWT Improved Intestinal Barrier Function in Mice with UC

**Figure 3:**
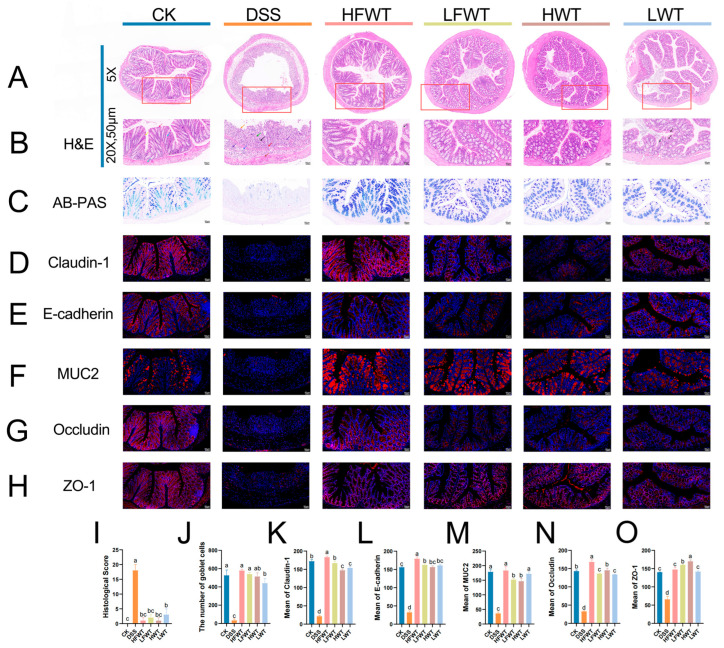
FWT enhanced intestinal barrier function in UC mice. (**A**,**B**) H&E-stained colon sections (The red boxes outline the areas that are shown at 20× magnification in the corresponding figures); (**C**) AB-PAS-stained colon sections; (**D**–**H**) immunofluorescence of claudin-1, E-cadherin, MUC2, occludin, and ZO-1; (**I**) histopathological score; (**J**) goblet cell count; (**K**–**O**) mean protein density of claudin-1, E-cadherin, MUC2, occludin, and ZO-1. Data are mean ± SEM (*n* = 5). Abbreviations are defined in Figure 2. Letters a–d indicate significant differences (*p <* 0.05).

H&E staining revealed intact colonic structure in the CK group, whereas the DSS group exhibited severe mucosal damage, epithelial ulceration, submucosal edema, and inflammatory cell infiltration. Treatment with HFWT, LFWT, HWT, or LWT markedly alleviated these pathological changes, showing restored goblet cells and minimal inflammation (Figure 3A,B). Consequently, all treatment groups significantly reduced the DSS-induced histopathological scores (*p <* 0.05, Figure 3I).

AB-PAS staining revealed that DSS treatment severely reduced goblet cells and degraded the mucin layer, which was significantly mitigated by HFWT, LFWT, HWT, and LWT interventions (Figure 3C,J). FWT groups demonstrated superior goblet cell restoration versus WT groups. Immunofluorescence (Figure 3D–H) showed that DSS significantly downregulated the expression of claudin-1, occludin, ZO-1, E-cadherin, and MUC2 (*p <* 0.05), while all treatments markedly upregulated these proteins (*p <* 0.05; Figure 3K–O). Collectively, the treatments, particularly HFWT, effectively repaired the intestinal mucosal barrier and maintained its integrity.

### 3.4. The Effect of FWT on the Serum Inflammatory Cytokines

**Figure 4:**
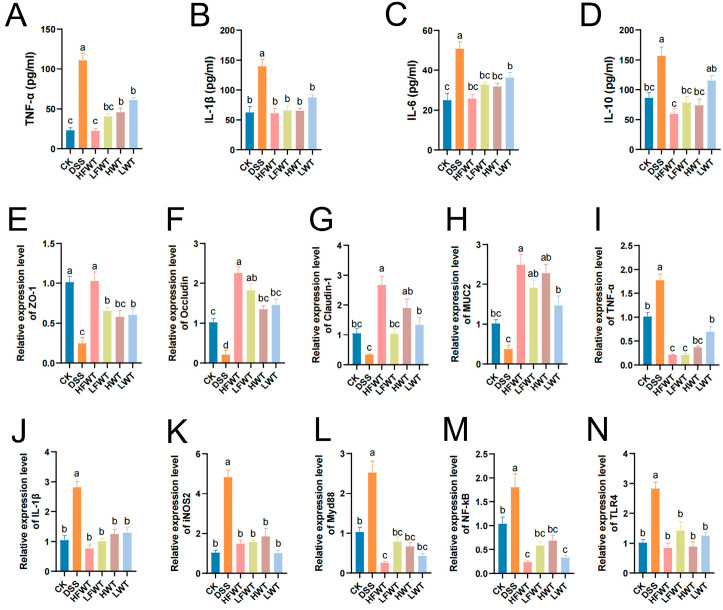
FWT modulates cytokine levels and gene expression. (**A**–**D**) Serum cytokines (TNF-α, IL-1β, IL-6, IL-10). (**E**–**N**) mRNA levels of barrier and immune-related genes (ZO-1, Occludin, Claudin-1, MUC-2, TNF-α, IL-1β, iNOS2, Myd88, NF-κB, TLR4). Data: mean ± SEM, *n* = 5. Letters denote significant differences (*p <* 0.05).

Serum levels of TNF-α, IL-1β, IL-6, and IL-10 were significantly elevated in the DSS group compared to the CK group (*p <* 0.05; Figure 4A–D). All treatment groups (HFWT, LFWT, HWT, LWT) significantly reduced these cytokine levels (*p <* 0.05), bringing them close to CK group levels. FWT was slightly more effective than WT, and higher doses outperformed lower doses, indicating a dose-dependent anti-inflammatory effect.

### 3.5. Regulation of FWT on the Expression of the Intestinal Mucosal Barrier and Immune-Related mRNA Gene

Next, qRT-PCR was performed to assess the mRNA levels of TJ proteins, MUC2, and inflammatory mediators. The DSS group showed significantly reduced expression of ZO-1, Occludin, Claudin-1, and MUC-2 (*p <* 0.05), whereas all FWT and WT treatments markedly increased their levels (Figure 4E–N), consistent with immunofluorescence results. Conversely, the expression of TNF-α, IL-1β, iNOS2, MyD88, NF-κB, and TLR4 was 1.78–4.84 times higher in the DSS group but significantly suppressed by FWT and WT treatments (*p <* 0.05). These findings indicate that FWT and WT enhance intestinal barrier integrity and inhibit key pro-inflammatory signaling (TLR4/MyD88/NF-κB), with HFWT exhibiting the most pronounced effects.

### 3.6. FWT Regulated the Production of SCFAs in Mice with UC

**Figure 5:**
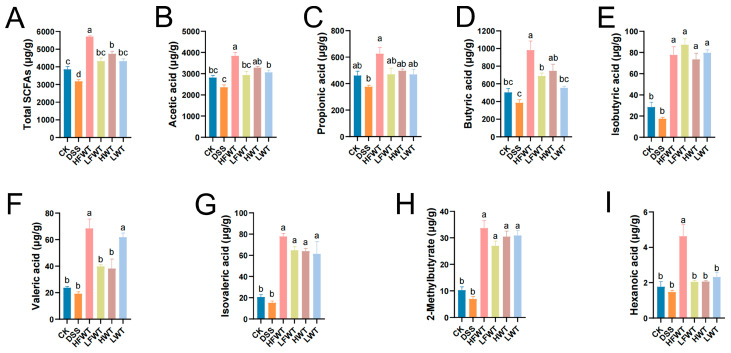
FWT alters SCFA profiles in UC mice. Quantified levels of (**A**) total SCFAs, (**B**) acetic, (**C**) propionic, (**D**) butyric, (**E**) isobutyric, (**F**) valeric, (**G**) isovaleric, (**H**) 2-methylbutyric, and (**I**) hexanoic acid. Data: mean ± SEM, *n* = 5. Letters indicate significance (*p <* 0.05).

SCFAs, such as acetic, propionic, and butyric acid, comprise over 90% of total SCFAs and are mainly produced by gut microbiota. As shown in Figure 5A–I, DSS treatment markedly reduced SCFA levels compared to the CK group. However, HFWT, LFWT, HWT, and LWT significantly reversed this decline (*p <* 0.05). HFWT showed the strongest effect, yielding the highest levels of butyric acid, caproic acid, and total SCFAs among all treated groups. These results indicate that both FWT and WT alleviate ulcerative colitis by modulating SCFAs, with HFWT being the most effective.

### 3.7. FWT Regulated the Gut Microbiota in DSS-Induced Mice

**Figure 6:**
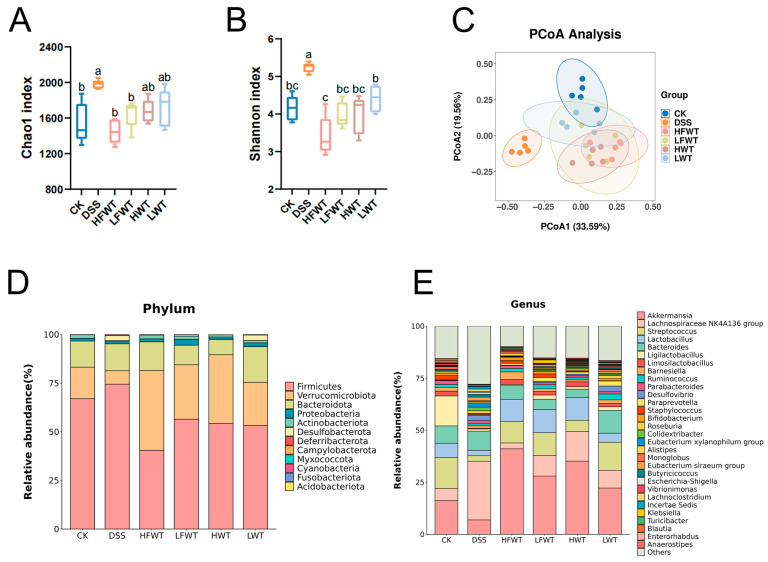
FWT ameliorates DSS-induced gut microbiota dysbiosis and restores community structure. (**A**) Chao index and (**B**) Shannon index, reflecting the α-diversity (richness and evenness) of the gut microbiota. (**C**) PCoA analysis (β-diversity) illustrating the overall structural separation of microbial communities among groups. (**D**) Stacked bar chart displaying the taxonomic composition at the phylum level for each group. (**E**) Stacked bar chart showing the relative abundance of the top 30 genera across all groups. Data are expressed as mean ± SEM (*n* = 5). Significant differences among groups are indicated by different letters (a–c, *p <* 0.05).

16S rRNA sequencing of colonic feces revealed that DSS administration significantly increased gut microbiota alpha-diversity (Chao1 and Shannon indices), which was subsequently normalized by HFWT, LFWT, HWT, and LWT treatments (Figure 6A,B). PCoA based on Bray–Curtis distance indicated clear separation between the DSS group and other groups, with partial overlap among the CK, LFWT, and LWT groups (Figure 6C), suggesting that both FWT and WT partially restored the DSS-disturbed microbiota structure.

At the phylum level, DSS increased the abundance of *Firmicutes* but decreased *Verrucomicrobiota* and *Actinobacteria*, while all treatments reversed these trends. HFWT showed the strongest effect, yielding the highest *Verrucomicrobiota* and lowest *Firmicutes* levels (Figure 6D and Figure S1A–E). Genus-level analysis indicated that DSS elevated *Lachnospiraceae NK4A136*_group and *Desulfovibrio* but reduced *Akkermansia*, *Streptococcus*, *Lactobacillus*, *Limosilactobacillus*, and *Bifidobacterium*. These alterations were most effectively reversed by HFWT, followed by LFWT and HWT, then LWT (Figure 6E and Figure S1F–K).

**Figure 7:**
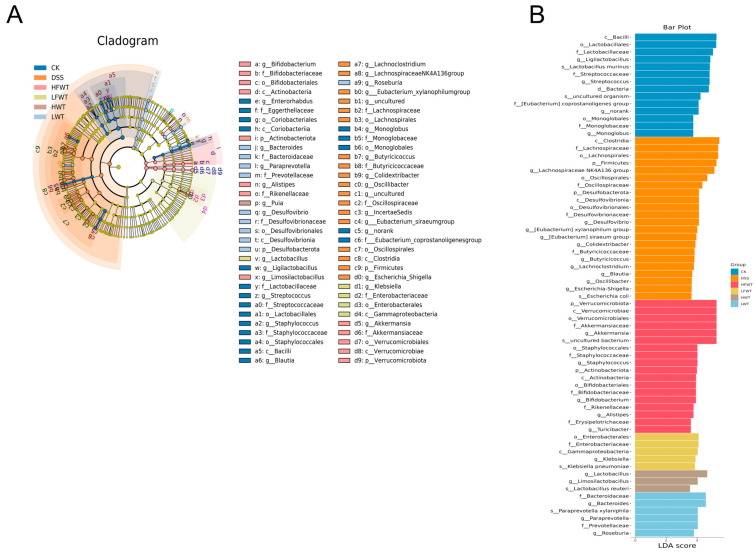
Identification of differentially enriched bacterial biomarkers through LEfSe analysis. (**A**) Cladogram generated by LEfSe analysis, depicting the phylogenetic distribution of microbial taxa with significant differences among groups. (**B**) Bar graph of the LDA scores (LDA > 3.5), highlighting the effect size of these discriminative features.

LEfSe analysis (LDA > 3.5) identified key gut microbiota alterations (Figure 7A,B). The DSS group was enriched in taxa, including the *Lachnospiraceae NK4A136* group, *Desulfovibrio*, and multiple lineages within *Firmicutes* and *Desulfobacterota*. In contrast, HFWT promoted beneficial probiotics such as *Akkermansia* and *Bifidobacterium*. LFWT was characterized by *Klebsiella*; HWT by *Lactobacillus* and *Limosilactobacillus*; and LWT by *Bacteroides*, *Paraprevotella*, and *Roseburia*. These results indicate that FWT and WT intervention was associated with modulation of the gut microbiota in DSS-induced mice, with HFWT showing the most pronounced increase in probiotics like *Akkermansia* and *Lactobacillus*.

### 3.8. FWT Regulated Colon Metabolites of DSS-Induced Mice

Fecal metabolomic profiling by UHPLC-MS identified 1604 metabolites, largely comprising lipids (21.07%), organic acids (14.03%), organoheterocyclic compounds (8.85%), and benzenoids (5.36%) (Figure S2). While principal component analysis (PCA) indicated some overlap of the DSS group with controls, orthogonal partial least squares discrimination analysis (OPLS-DA) demonstrated a distinct clustering of the DSS group apart from all other groups (Figure S3A,B).

**Figure 8:**
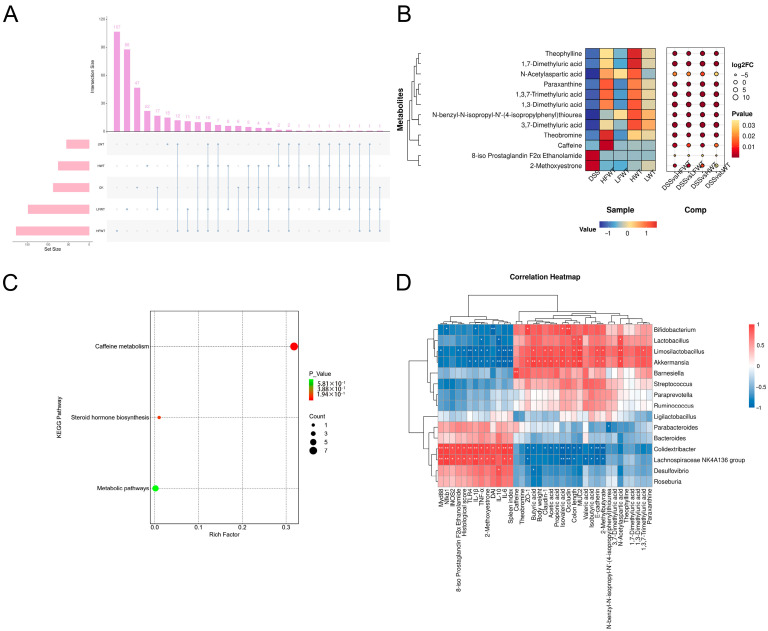
Functional analysis of differential metabolites and correlation with gut microbiota in UC mice. (**A**) Upset plot visualizing intersections of differential metabolites across comparisons. (**B**) Heatmap bubble chart depicting the relative abundance patterns of 12 key differential metabolites. (**C**) KEGG pathway enrichment analysis of identified differential metabolites. (**D**) Pearson correlation analysis between the top 30 gut microbiota genera and UC-related indicators. Asterisks denote significant correlations (* *p <* 0.05, ** *p <* 0.01). Data are presented as mean ± SEM (*n* = 5).

Volcano plots (Figure S3C) revealed 90 differential metabolites between the CK and DSS groups (40 up, 50 down; |log_2_FC| > 1, VIP > 1, *p <* 0.05). Relative to the DSS group, the HFWT, LFWT, HWT, and LWT groups exhibited 177 (133 up, 44 down), 148 (130 up, 18 down), 75 (45 up, 30 down), and 55 (41 up, 14 down) differentially abundant metabolites, respectively. Upset plot analysis identified 12 common differential metabolites across all treatment groups compared to DSS (Figure 8A), visualized in a heatmap/bubble plot (Figure 8B). Among these, 8-iso Prostaglandin F2α Ethanolamide and 2-Methoxyestrone were downregulated in all treatment groups. In contrast, caffeine, theobromine, paraxanthine, and 1,3,7-trimethyluric acid were highest in HFWT, while theophylline and three dimethyluric acids were highest in HWT. KEGG enrichment analysis indicated these metabolites were significantly associated with caffeine metabolism and steroid hormone biosynthesis (Figure 8C).

### 3.9. Pearson Correlation Analysis Between Gut Microbiota and UC Indicators

A correlation analysis was conducted among the top 15 abundant genera of gut microbiota and indicators related to colitis. As illustrated in Figure 8D, four notably beneficial bacteria—*Akkermansia*, *Lactobacillus*, *Bifidobacterium*, and *Limosilactobacillus*—were positively correlated with the production of SCFAs, the activation of caffeine pathways, and intestinal barrier function. In contrast, these genera exhibited negative correlations with colon lesions, the activation of inflammatory pathways, and the production of inflammatory factors. Conversely, the *Lachnospiraceae NK4A136* group and *Colidextribacter* demonstrated opposite correlations compared to the four aforementioned genera.

### 3.10. Single-Cell Sequencing Analysis Revealed That FWT Intervention Significantly Reshaped the Cellular Landscape in Colitis Mice

**Figure 9:**
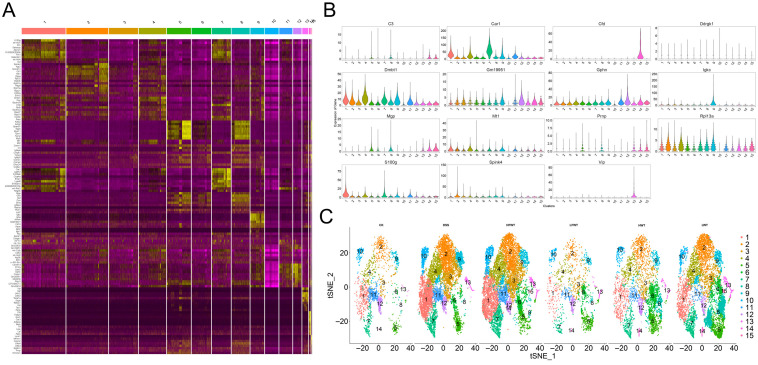
FWT intervention significantly reshaped the cellular landscape in colitis mice, revealing distinct cell clustering and classification patterns. (**A**) Heatmap displaying the non-redundant top 10 marker genes per cell cluster. Columns correspond to individual clusters (separated by gaps), and rows represent cluster-specific markers, with expression levels color-coded from yellow (high) to purple (low). (**B**) Violin plots illustrating the expression distribution of key marker genes across identified clusters. (**C**) t-SNE visualization based on unsupervised random forest classification, depicting the distribution of filtered cells from each experimental group (CK, DSS, HFWT, LFWT, HWT, LWT).

Single-cell RNA sequencing analysis revealed that FWT intervention significantly remodeled cellular heterogeneity in colitis-afflicted mice, with unsupervised clustering identifying 15 functionally distinct subpopulations characterized by cluster-specific enrichment of key marker genes: carbonic anhydrase 1, (CAR1, encoding carbonic anhydrase 1) was enriched in cluster 7, while complement factor D (CFD, an inflammatory response biomarker) exhibited marked upregulation in cluster 14. Notably, elevated expression patterns were observed for Deleted in Malignant Brain Tumors 1 (DMBT1, a tumor suppressor) in cluster 4, the long non-coding RNA GM19951 in cluster 11, Matrix Gla Protein (MGP, a multifunctional vitamin K-dependent protein) in cluster 8, metallothionein 1 (MT1, functioning in metal transport and antioxidation) in cluster 5, Ribosomal Protein L13a (RPL13A) in cluster 3, S100 Calcium Binding Protein G (S100G, a calcium-binding protein family member) in cluster 1, and vasoactive intestinal peptide (VIP, an immunoregulatory neuropeptide) in cluster 13. Clusters #1–4, 7, and 9–12 were predominantly localized in DSS and HFWT groups with substantial representation in LWT (Figure 9A–C), whereas clusters #5, 8, and 14 were primarily enriched in the LWT group.

**Figure 10:**
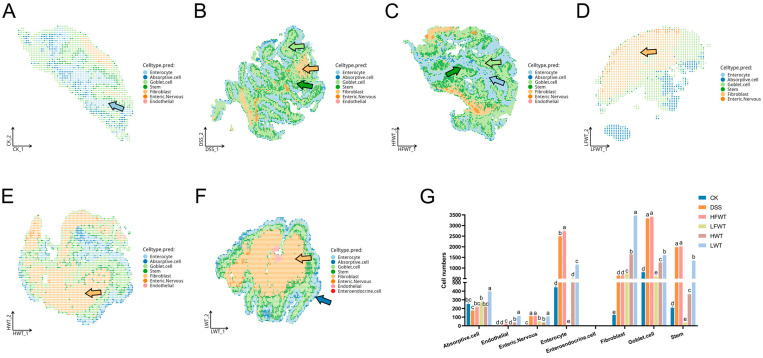
FWT intervention altered cell-type composition and distribution in colitis mice. (**A**–**F**) Single-cell transcriptional annotation maps visualized in a shared embedding space for each experimental group. Arrows indicate annotated cell types: light blue = enterocytes, dark blue = absorptive cells, light green = goblet cells, dark green = stem cells, yellow = fibroblasts. (**G**) Quantitative analysis of the proportion of different cell types per sample. Data are presented as mean ± SEM (*n* = 3). Significant differences among groups are indicated by letters a–e (*p <* 0.05).

Spatial transcriptomics precisely delineated the in situ distribution patterns of cells (Figure 10A–F). In the LWT group, absorptive cells formed high-density functional zones at the villus apex (397 cells/mm^2^), achieving a density 2.27-fold higher than that in the DSS group. This indicates an accelerated recovery of colonic nutrient absorption function. Continuous secretory bands formed by goblet cells within the crypt region, along with a significant increase in the number of stem cells at the crypt base, were observed in both the DSS and HFWT groups. This confirms the involvement of stem cell activation in the regenerative process. Notably, despite the presence of compensatory repair in the DSS group, it exhibited disruption of the villus–crypt axis architecture. This was characterized by the aberrant migration of goblet cell precursors into the superficial villus epithelium. In contrast, the HFWT group not only exhibited an increased number of goblet cells but also demonstrated significantly higher MUC2 mucin secretion compared to the DSS group. This collectively indicates that HFWT facilitates effective barrier reconstruction by restoring the order of stem cell differentiation, optimizing goblet cell functionality, and ameliorating the microenvironment.

Cell population dynamics analysis revealed that compared to the normal control group (CK), the DSS model group exhibited a significant increase (*p <* 0.05) in the number of enteric nervous, enterocytes, fibroblasts, goblet cells, and stem cells. Conversely, the number of absorptive cells was reduced. This pattern reflects an imbalance between compensatory repair mechanisms and ongoing tissue injury. In the LFWT, HWT, and LWT groups, the numbers of enterocytes, goblet cells, and stem cells were significantly lower (*p <* 0.05) than those in the DSS group. However, the fibroblast population showed a significant increase (*p <* 0.05). This suggests that these treatments may promote fibroblast-mediated microenvironmental remodeling to support epithelial repair (Figure 10G).

### 3.11. Single-Cell and Spatial Transcriptomic Profiling Reveals Dose-Dependent Modulation of Cell-Specific Gene Networks and Neuro–Immune–Stromal Crosstalk in DSS-Induced Colitis by FWT

scRNA-seq analysis demonstrated that DSS-induced colitis triggered significant cell-type-dependent transcriptional dysregulation (Figure S4A). In absorptive cells, expression of the zinc-α2-glycoprotein gene ZG16 and the calcium-activated chloride channel regulator CLCA1 was significantly upregulated, while expression of the interleukin-31 receptor α subunit IL31RA and the long non-coding RNA GM42418 was downregulated, suggesting an enhancement of intestinal barrier function to counteract inflammatory damage. Enterocytes exhibited elevated expression of ZG16 and the calcium-binding protein S100G alongside suppressed expression of IL31RA and the pyroptosis-associated protein GSDMC4, collectively contributing to maintaining calcium homeostasis and blocking the epithelial pyroptosis pathway. Fibroblasts showed increased transcript levels of carbonic anhydrase CAR1 and the anchoring protein GPHN concurrent with decreased expression of smooth muscle actin isoforms ACTG2 and ACTA2, indicating suppression of fibrotic processes and enhanced tissue regenerative capacity. Goblet cells displayed significant upregulation of the immunoglobulin heavy chain IGHA, κ light chain IGKC, GPHN, and ZG16 coupled with downregulated expression of the secreted glycoprotein AGR2 and λ light chain IGLC1, reflecting a coordinated optimization mechanism for the mucosal immune barrier. Stem cells demonstrated increased expression of the RNA modification-associated gene SNORD13, ZG16, and the rRNA processing gene SNORD118 with decreased expression of GM42418 and the calmodulin kinase CAMK1D, synergistically acting to maintain stem cell homeostasis.

HFWT intervention reversed the progression of colitis through transcellular synergistic mechanisms (Figure S4B). Barrier repair genes such as regenerating family member 3 beta (REG3B) were broadly upregulated in absorptive cells, fibroblasts, and stem cells, while expression of the antibacterial factor LY6/PLAUR domain containing 8 (LYPD8) was synchronously elevated in enterocytes, goblet cells, and stem cells, collectively promoting intestinal barrier restoration and inflammation suppression. Notably, the long non-coding RNA GM42418 exhibited consistent upregulation across absorptive cells, enterocytes, fibroblasts, and stem cells, suggesting its potential role as a novel immune–stromal regulator. In endothelial cells, expression of the C-C motif chemokine ligand 6 (CCL6) and the ribosomal protein lateral stalk subunit P1 pseudogene 1 (RPLP1) was significantly suppressed. Conversely, the enteric nervous system demonstrated coordinated upregulation of somatostatin (SST), myosin heavy chain 11 (MYH11), tachykinin precursor 1 (TAC1), and galanin (GAL). Goblet cells displayed synergistic high expression of immunoglobulin kappa constant (IGKC), immunoglobulin lambda constant 1 (IGLC1), ZG16, and S100G, concurrently with transcriptional downregulation of the small nucleolar RNAs SNORD13 and SNORD118.

Following LFWT treatment, absorptive cells exhibited significantly enhanced expression of the regenerating islet-derived proteins regenerating family member 3 gamma (REG3G) and REG3B, along with the GM42418. Concurrently, the enteric nervous system demonstrated synchronous upregulation of TAC1, proenkephalin (PENK), and SST. A consistent downregulation of carbonic anhydrase CAR1 was detected across multiple cell types, including absorptive cells, enterocytes, fibroblasts, goblet cells, and stem cells. Furthermore, synchronously suppressed expression of immunoglobulin heavy constant alpha (IGHA) and IGKC was observed specifically within fibroblasts, goblet cells, and stem cells (Figure S4C).

Following HWT intervention, absorptive cells exhibited upregulated expression of REG3B, GM42418, and LYPD8. Endothelial cells displayed significantly elevated levels of complement factor D (CFD), fatty acid binding protein 4 (FABP4), and carbonic anhydrase 3 (CAR3). The enteric nervous system showed increased expression of SST and neuromedin U (NMU) alongside decreased expression of VIP and calcium/calmodulin-dependent protein kinase ID (CAMK1D). Expression of the regenerating protein regenerating family member 4 (REG4) was broadly upregulated in enterocytes, fibroblasts, and stem cells. Enterocytes specifically demonstrated elevated expression of peroxiredoxin 6 (PRDX6), concurrent with suppressed expression of CAR1. Fibroblasts and goblet cells exhibited downregulated expression of IGHA and IGKC, while absorptive cells, enterocytes, goblet cells, and stem cells displayed synchronous upregulation of serine peptidase inhibitor Kazal type 4 (SPINK4) (Figure S4D).

LWT treatment induced significantly enhanced expression of S100G, GM42418, and CAR1 in absorptive cells (*p <* 0.05). Endothelial cells exhibited simultaneous elevation of CFD, FABP4, and stearoyl-CoA desaturase 1 (SCD1). Within the enteric nervous system, significant upregulation of galanin (GAL), VIP, and TAC1 was observed. Enterocytes displayed increased expression of peroxiredoxin 6 (PRDX6) and SPINK4 concurrently with decreased expression of CAR1. Fibroblasts demonstrated elevated expression of CAR1, SPINK4, and ACTG2, while expression of CAMK1D, IGKC, and cyclin-dependent kinase 8 (CDK8) was suppressed. Goblet cells showed downregulated expression of IGHA and IGKC, whereas stem cells exhibited synergistic upregulation of REG4, CAR1, and SPINK4 (Figure S4E).

## 4. Discussion

UC is a chronic condition marked by diarrhea, abdominal pain, weight loss, and sometimes intestinal bleeding, which elevates cancer risk. Current UC therapies often cause severe side effects, underscoring the need for safer treatments [29]. While tea and its components have shown anti-colitis potential, the role of FWT in UC remains unclear. This study evaluated the effects of FWT on UC. Results showed that FWT alleviated DSS-induced colon injury, restored mucosal barrier and immune function, reshaped gut microbiota, increased beneficial bacteria, and elevated SCFAs and caffeine pathway metabolites. Integrated single-cell RNA sequencing and spatial transcriptomics revealed that HFWT increased enterocytes, goblet cells, and stem cells, enhancing the mucus barrier via MUC2 secretion and normalized stem cell differentiation. In contrast, LFWT expanded fibroblasts, aiding tissue repair. Furthermore, FWT modulated cell-specific gene networks (REG3B, LYPD8, GM42418) and neuro–immune–stromal crosstalk, remodeling cellular heterogeneity and promoting barrier repair, anti-inflammation, and tissue regeneration. Overall, HFWT demonstrated superior efficacy in alleviating colitis.

The significant alterations in the phytochemical profile of FWT, including the marked decrease in tea polyphenols, free amino acids, and soluble sugars alongside an increase in caffeine, are consistent with the known metabolic activities of *Eurotium cristatum*. These changes likely result from the fungal utilization of amino acids and sugars as nitrogen and carbon sources, the relative stability or potential biosynthesis of caffeine, and the enzymatic hydrolysis of esterified catechins [30,31].

In this study, both FWT and WT reversed DSS-induced weight loss, bloody stools, elevated DAI scores, spleen index increase, and colon shortening in mice. Colon pathology further showed that they ameliorated crypt structure loss, reduction in epithelial and goblet cells, intestinal mucosal edema, and inflammatory cell infiltration into the muscularis mucosa. These results indicated that FWT and WT alleviated UC, consistent with previous studies on tea and its active components [32]. Moreover, FWT exerted a stronger therapeutic effect than WT in a dose-dependent manner.

The intestinal mucosal barrier, critical for intestinal function, relies on TJ proteins (restricting paracellular transport), MUC2 (forming a protective mucus layer), and E-cadherin (maintaining epithelial integrity) [33,34,35]. In UC, their dysfunction compromises the barrier, enabling bacterial translocation and inflammation. This study found that FWT and WT enhanced TJ protein, MUC2, and E-cadherin expression (immunofluorescence) and reduced intestinal permeability. Correspondingly, qPCR showed increased mRNA levels of ZO-1, occludin, claudin-1, and MUC2, indicating that FWT reinforces the barrier [36]. Single-cell sequencing revealed DSS-induced inflammation triggers epithelial reprogramming: absorptive cells upregulated barrier genes (ZG16, CLCA1) but downregulated IL31RA, suppressed GSDMC4 (preventing pyroptosis), and upregulated S100G (calcium homeostasis) [37,38,39,40]. Fibroblasts downregulated fibrotic markers (ACTG2/ACTA2) and activated regeneration gene GPHN [41,42,43], while goblet cells suppressed AGR2 (impairing mucus secretion). Post-FWT intervention, absorptive cells significantly upregulated barrier-repair genes REG3B and LYPD8 [44,45]. Spatial transcriptomics further showed that HFWT promoted continuous goblet cell secretory bands in crypts and increased crypt-base stem cells. Thus, single-cell and spatial transcriptomic data suggest that FWT may remodel cellular heterogeneity through the modulation of cell-specific gene networks, which could facilitate barrier repair and tissue regeneration

TLR4, a pattern recognition receptor, initiates colitis-associated inflammation upon recognizing intestinal pathogens. Ligand binding promotes TLR4-Myd88 interaction and activates NF-κB signaling, leading to nuclear translocation and transcription of pro-inflammatory cytokines (TNF-α, IL-1β, IL-6). These cytokines amplify immune activation, recruit leukocytes, disrupt epithelial integrity, and exacerbate inflammation [46,47]. Inflammatory conditions also upregulate iNOS2, resulting in excessive NO and tissue-damaging peroxynitrite [48]. In this study, DSS elevated IL-6, TNF-α, IL-1β, and IL-10 levels, while both FWT and WT significantly suppressed these cytokines. The observed IL-10 increase in DSS groups, which was contrary to Wei et al. [49], may reflect a compensatory anti-inflammatory response during sustained immune activation. IL-10 is renowned for its dual role in immune regulation, acting as a central brake on excessive inflammation [50]. In the context of active colitis, its upregulation likely represents the host’s attempt to counterbalance the overwhelming production of pro-inflammatory cytokines (e.g., TNF-α, IL-6) and to restrain overactive immune cells [51]. Mechanistically, IL-10 exerts its anti-inflammatory effects partly by reprogramming macrophage metabolism, such as inhibiting glycolytic activity and promoting mitochondrial health, thereby suppressing the production of inflammatory mediators [52]. Therefore, the reduction in IL-10 levels by FWT intervention is not indicative of suppressing a beneficial signal but rather suggests that FWT may so effectively dampen the upstream drivers of inflammation (e.g., TLR4/NF-κB pathway) and ameliorate tissue damage that the need for such a heightened compensatory IL-10 response is diminished. This shift might signify a restoration of immune homeostasis, where the sustained, high-level ‘emergency’ anti-inflammatory signaling is no longer required and is a mechanism requiring further investigation. qRT-PCR confirmed that FWT downregulated TNF-α, IL-1β, IL-6, iNOS2, TLR4, Myd88, and NF-κB mRNA in colon tissues, consistent with reported anti-colitic effects of tea components [53,54]. Thus, FWT alleviates colitis by inhibiting inflammatory factors and signaling pathways, with HFWT being most effective.

SCFAs have multiple roles in colitis treatment. They improve intestinal barrier integrity by promoting mucus secretion and enhancing epithelial tight junctions. SCFAs also regulate intestinal immunity, boost immune cell activity, inhibit the NF-κB pathway, and reduce inflammatory factor release. Additionally, as energy substrates for enterocytes, SCFAs promote cell proliferation/repair and alleviate inflammation [55,56]. Primary SCFAs produced by beneficial bacteria (such as *Akkermansia*, *Bifidobacterium* and *Lactobacillus*) ferment dietary fiber [57]. Compared to DSS, FWT groups significantly increased SCFA concentrations, notably butyric acid and isobutyric acid. Butyric acid specifically has therapeutic effects in UC, enhancing barrier function and mucin secretion [58]. The greatest SCFAs increase in HFWT may relate to its significant enrichment of *Akkermansia*, *Lactobacillus*, and *Bifidobacterium*.

Gut microbiota dysbiosis is crucial in UC pathogenesis [59]. Contrary to most studies [60,61], DSS treatment in this study increased microbial diversity and richness, potentially due to elevated intestinal permeability and harmful bacterial proliferation [62]. FWT treatments reduced harmful bacteria and normalized abundance and diversity versus the DSS group. At the phylum level, DSS decreased beneficial *Verrucomicrobiota* and *Actinobacteria* but increased *Firmicutes* (which contains harmful bacteria). At the genus level, DSS reduced beneficial *Akkermansia*, *Lactobacillus*, and *Bifidobacterium* while increasing the harmful *Lachnospiraceae NK4A136* group, *Desulfovibrio*, and *Colidextribacter* [63,64,65]. FWT counteracted this trend, with HFWT showing the most pronounced effect. *Akkermansia*, *Lactobacillus*, and *Bifidobacterium* are probiotics that enhance tight junction protein expression, regulate immune cell activity, modulate inflammatory factors, and promote beneficial flora growth [66,67,68]. *Eurotium cristatum* metabolites can increase probiotics such as *Bifidobacterium* [69]. LEfSe analysis confirmed that HFWT significantly increased *Akkermansia* and *Bifidobacterium* abundances. Thus, FWT alleviated DSS-induced dysbiosis and enriched SCFA-producing beneficial flora, with HFWT being most effective.

DSS treatment altered metabolite profiles. Compared to DSS, the HFWT, LFWT, HWT, and LWT groups exhibited 12 differential metabolites. DSS showed the highest levels of 8-iso Prostaglandin F2α Ethanolamide and 2-Methoxyestrone, indicating oxidative damage in UC pathogenesis [70,71]. HFWT contained the highest concentrations of caffeine, theobromine, paraxanthine, and 1,3,7-trimethyluric acid, while HWT had the highest levels of theophylline, 1,3-dimethyluric acid, 3,7-dimethyluric acid, and 1,7-dimethyluric acid. Caffeine regulates metabolism with anti-inflammatory and antioxidant effects [72]. Theobromine boosts SCFAs and benefits intestinal health [73]. Elevated 1,3,7-trimethyluric acid, an intermediate in caffeine metabolism, suggests an altered profile of caffeine metabolism, which may involve contributions from gut microbiota. *Eurotium cristatum* fermentation increased caffeine content in FWT, potentially inhibiting conversion to theophylline while favoring theobromine and 1,3,7-trimethyluric acid production. Caffeine can increase *Bifidobacterium* abundance, maintaining microbiota balance and immunity. *Lactobacillus* and *Bifidobacterium* influence caffeine metabolism through pH changes or enzyme activity [74,75,76]. *Akkermansia*, *Lactobacillus*, and *Bifidobacterium* promote SCFAs, enhance barrier function via tight junction proteins, and inhibit inflammation. Pearson correlations confirmed these genera positively correlate with SCFAs, caffeine metabolism, and barrier function but negatively correlate with inflammation and lesions. It is important to note that the elevated levels of caffeine-derived metabolites in feces could reflect enhanced microbial metabolism, altered host hepatic processing, or reduced intestinal absorption. While the positive correlation with specific probiotics supports a potential microbial role, future metagenomic or enzymatic studies are needed to definitively assign this metabolic function to specific taxa and disentangle host versus microbial contributions.

Furthermore, the regulatory role of FWT on intestinal neuro–immune–stromal interactions warrants in-depth investigation. Single-cell sequencing results demonstrated that FWT intervention significantly upregulated the expression of genes within the enteric nervous system, including SST, MYH11, TAC1, and GAL. These changes are closely associated with enteric nervous system activation, which plays a critical role in modulating intestinal immune responses and tissue repair [77,78,79]. For instance, the upregulation of TAC1 may promote neurotransmitter release and modulate inflammatory responses in the gut [80]. Integrating these findings with spatial transcriptomics observations of a significant increase in fibroblast numbers within the LFWT group suggests that FWT likely promotes fibroblast-mediated microenvironment remodeling. This remodeling supports epithelial repair and reinforces intestinal barrier function. This cross-cellular synergy provides new insights into how FWT alleviates UC through neuro–immune–stromal interactions. However, whether these microbial and metabolic changes are necessary mediators for the therapeutic effects of FWT remains to be experimentally confirmed. Furthermore, the immune-modulatory mechanisms inferred from transcriptomic data require validation at the protein and cellular level. Future studies should therefore integrate causal approaches, such as antibiotic-mediated microbiota depletion or fecal microbiota transplantation, with direct immune phenotyping (flow cytometry) to definitively decipher the mechanisms of FWT and to complement the transcriptional insights provided by snRNA-seq, which, while powerful, does not fully resolve specific immune cell subtypes.

## 5. Conclusions

In summary, FWT contained higher caffeine but lower tea polyphenols and free amino acids than WT. It suppressed inflammatory signaling (TLR4/Myd88/NF-κB) and cytokines (TNF-α/IL-6/IL-1β) while enhancing gut barrier integrity via TJ proteins, MUC2, and E-cadherin. Single-cell and spatial transcriptomics showed HFWT increased enterocytes, goblet cells, and stem cells, while LFWT expanded fibroblasts. HFWT established an “antimicrobial-barrier-immunity” network by upregulating REG3B (binds peptidoglycan), LYPD8 (inhibits flagellin), and IGHA/IGKC (enhances mucosal immunity). The lncRNA GM42418, upregulated across multiple cell types and reversed by DSS, emerged as a potential key regulator and represents a candidate for further investigation as a therapeutic target. Furthermore, FWT intervention resulted in a modulated microbiota–metabolite axis, characterized by enrichment of *Akkermansia*, *Lactobacillus*, and *Bifidobacterium*, elevated SCFAs, and altered caffeine metabolism, which collectively correlated with reduced inflammation. These results support FWT as a promising adjunctive candidate for UC. While this multi-scale study delineates the integrative effects of FWT, its complex nature presents challenges in attributing benefits to single compounds, and the causal links within the microbiota–metabolite–immunity axis remain to be fully deciphered. Future efforts should therefore focus on pinpointing the active constituents, employing causal models like fecal microbiota transplantation to verify mechanisms, and ultimately translating these findings into clinical evaluation.

## Figures and Tables

**Table 1:** The Disease Activity Index scale.

Score	Weight Loss	Stool Consistency	Rectal Bleeding
0	None	Normal	Normal
1	1–5%	Loose stool	Occult bleeding
2	5–10%	Slight diarrhea	Slight bleeding
3	10–20%	Gross diarrhea	Obvious bleeding
4	>20%	Watery diarrhea	Gross bleeding

**Table 2:** Primer sequences.

Name	Forward Primer (5′->3′)	Reverse Primer (5′->3′)
GAPDH	TGGAAAGCTGTGGCGTGATG	TACTTGGCAGGTTTCTCCAGG
MUC2	TCCCGACTTCAACCCAAGTG	TGGGCAGAGTACATGGCAAA
occludin	TGCTTCATCGCTTCCTTAGTAA	GGGTTCACTCCCATTATGTACA
claudin-1	GCCTTGATGGTAATTGGCATCC	GGCCACTAATGTCGCCAGAC
TNF-α	CGTCGTAGCAAACCACCAAG	GGCAGAGAGGAGGTTGACTT
IL-1β	TCAGGCAGGCAGTATCACTC	TTGTTCATCTCGGAGCCTGT
iNOS2	GAAGGGGACGAACTCAGTGG	GTGGCTCCCATGTTGCATTG
TLR4	CTTGAATCCCTGCATAGAGGTAG	AGCTCAGATCTATGTTCTTGGTTGA
ZO-1	CTGGTGAAGTCTCGGAAAAATG	CATCTCTTGCTGCCAAACTATC
Myd88	GCAACCTTCAGTGCCAGAGA	CCAGGTAGAGCCACAGACCA
NF-kB	GCTGCCAAAGAAGGACACGACA	GGCAGGCTATTGCTCATCACAG

**Table 3:** The contents of water, water extracts, tea polyphenols, free amino acid, soluble sugar, and caffeine in FWT and WT extracts. The different letters represent significant differences among each group (*p <* 0.05).

Constituent	FWT	WT
Water (%)	10.03 ± 0.386 ^a^	7.50 ± 0.734 ^b^
Water extracts (%)	35.77 ± 0.695 ^b^	37.3 ± 0.486 ^a^
Tea polyphenols (%)	6.94 ± 0.207 ^b^	7.89 ± 0.312 ^a^
Free amino acid (%)	3.40 ± 0.150 ^b^	6.88 ± 0.093 ^a^
Soluble sugar (%)	3.76 ± 0.350	4.51 ± 0.431
Caffeine (%)	2.95 ± 0.150 ^a^	2.56 ± 0.093 ^b^

## Data Availability

The original contributions presented in this study are included in the article/Supplementary Materials; further inquiries can be directed to the corresponding authors.

## Supplementary Materials

The following supporting information can be downloaded at https://www.mdpi.com/article/10.3390/foods15010072/s1. Table S1. Detailed information on metabolites identified in this study. Figure S1: FWT modulates the abundance of specific bacterial taxa at both the phylum and genus levels. (A–E) Bar plots demonstrating the relative abundance changes of five representative bacterial phyla. (F–K) Bar plots demonstrating the relative abundance changes of six representative bacterial genera. Data are expressed as mean ± SEM (*n* = 5). Significant differences among groups are indicated by different letters (a–d, *p <* 0.05). Figure S2: Metabolite classification pie chart. Figure S3: FWT alters the colon fecal metabolic profile in DSS-induced UC mice. (A) PCA score plot illustrating overall metabolic variations among experimental groups. (B) OPLS-DA score plot highlighting group separations and identifying key metabolic contributors. (C) Volcano plot displaying significantly altered metabolites based on thresholds (|log_2_FC| > 1, VIP > 1, and *p <* 0.05). Figures S4–S8 present volcano plots of differentially expressed genes (|log_2_FC| > 1, *p <* 0.05) for the pairwise comparisons of DSS vs. CK, HFWT vs. DSS, LFWT vs. DSS, HWT vs. DSS, and LWT vs. DSS, respectively.
