# Supplementary figures and images for: Eurotium cristatum-Fermented White Tea Ameliorates DSS-Induced Colitis by Multi-Scale
Source: Foods. 2025 Dec 25;15(1):72. doi: 10.3390/foods15010072 (PMC12786079; doi:10.3390/foods15010072)

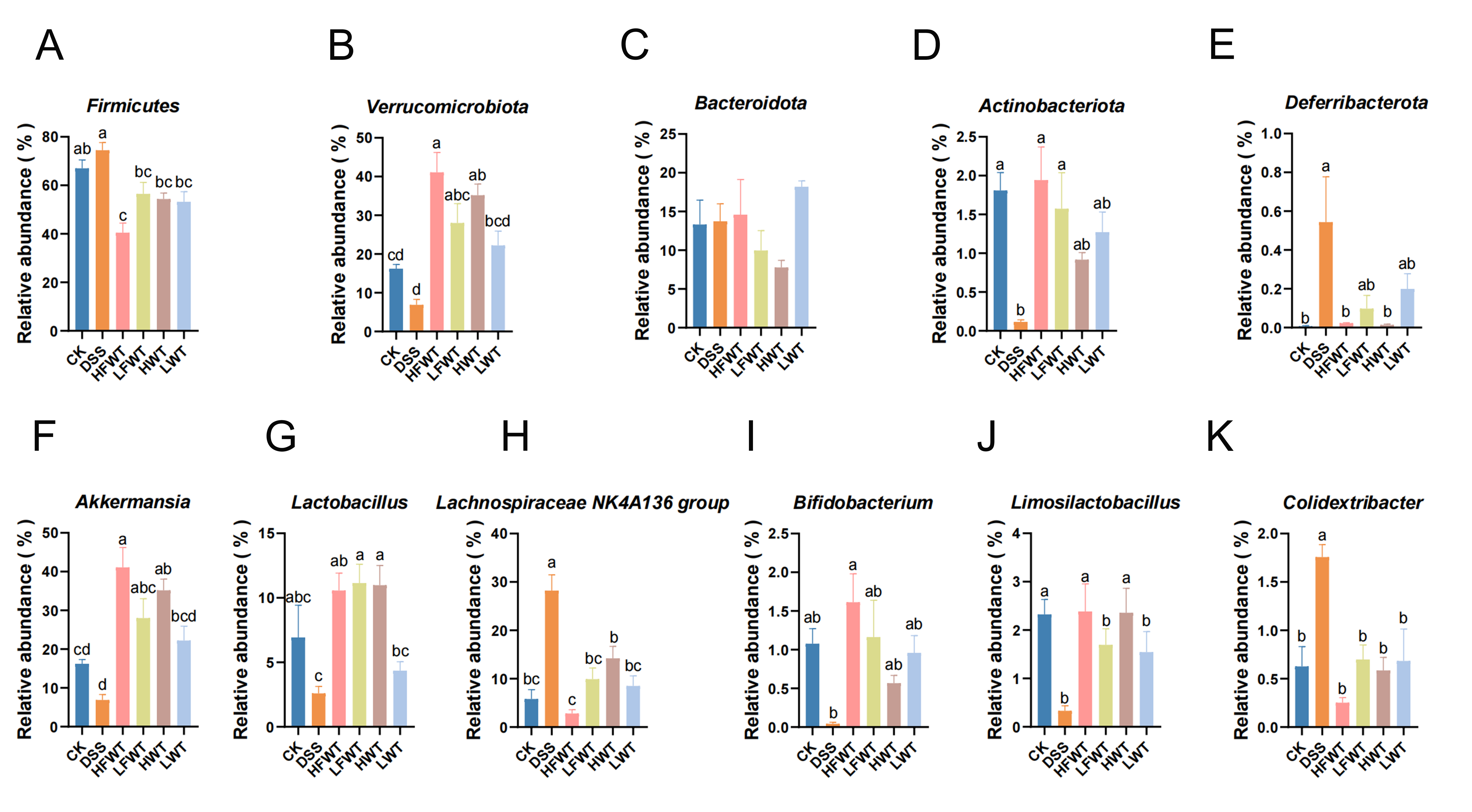

Supplement: Supplementary file 1 [file foods-15-00072-s001.zip › Fig. S1.png]

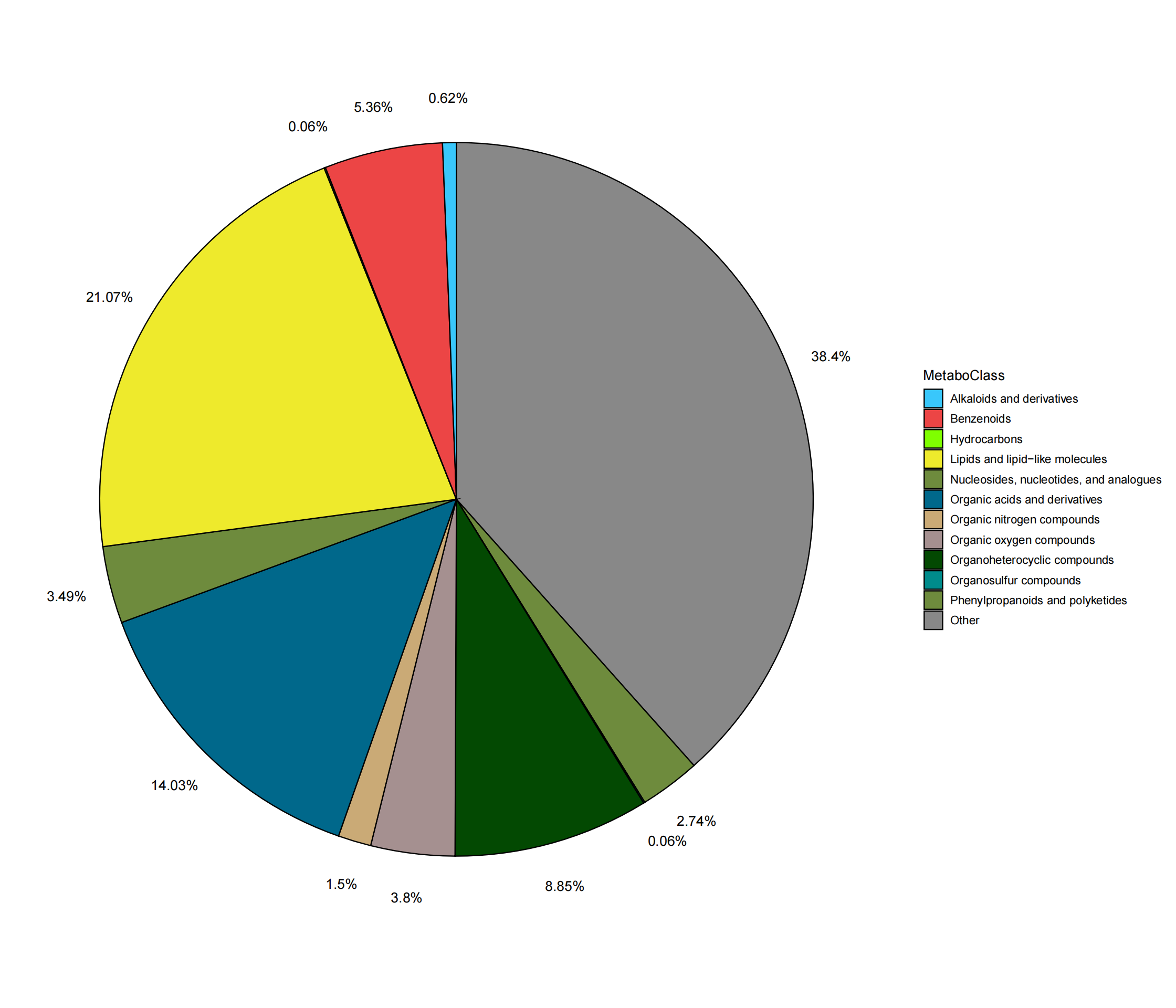

Supplement: Supplementary file 1 [file foods-15-00072-s001.zip › Fig. S2.png]

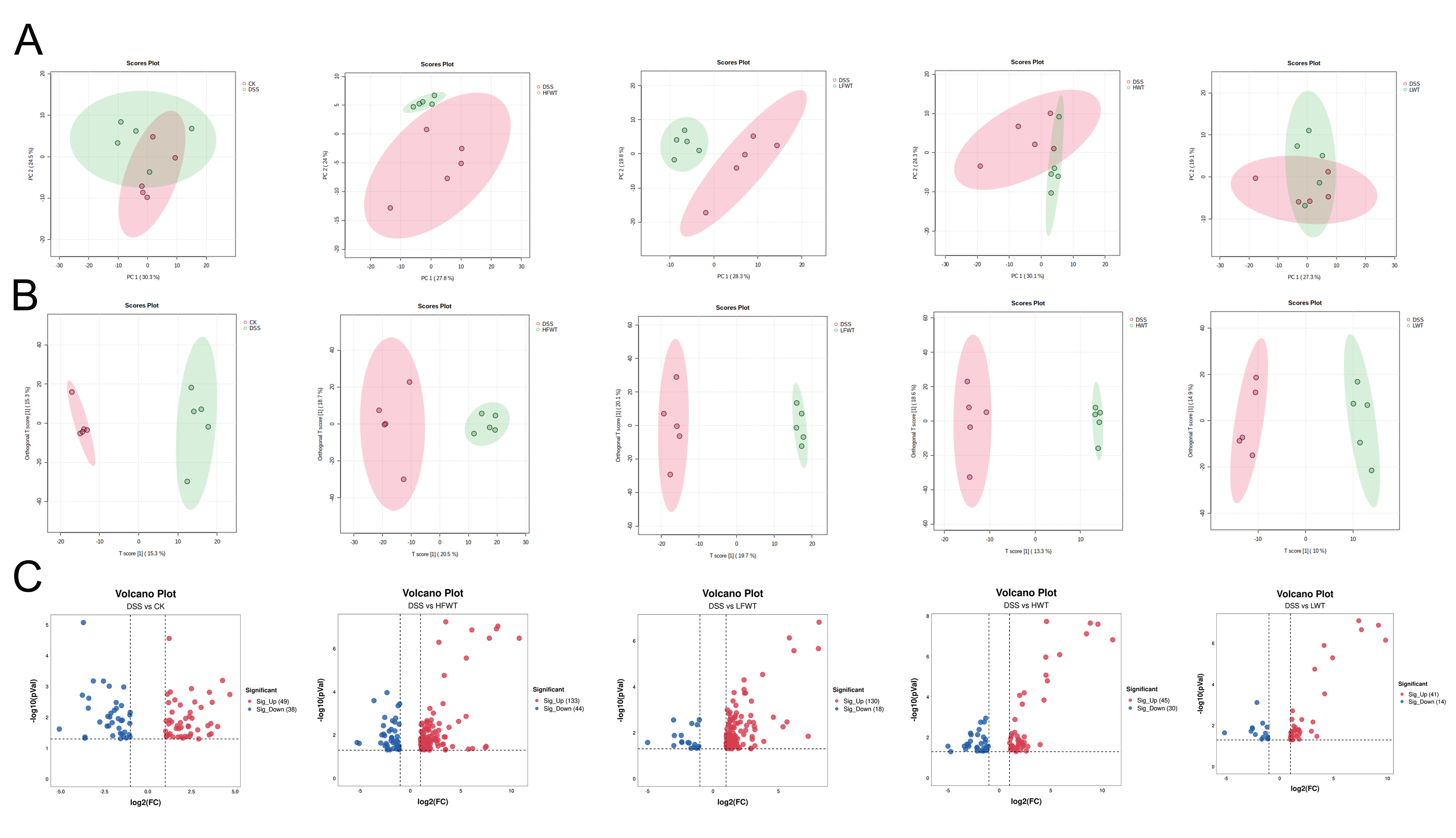

Supplement: Supplementary file 1 [file foods-15-00072-s001.zip › Fig. S3.png]
